# The role of the *REG4* gene and its encoding product in ovarian epithelial carcinoma

**DOI:** 10.1186/s12885-015-1435-2

**Published:** 2015-06-16

**Authors:** Shuo Chen, Wen-Feng Gou, Shuang Zhao, Zhe-Feng Niu, Yang Zhao, Yasuo Takano, Hua-Chuan Zheng

**Affiliations:** 1 Department of Gynecology, The First Affiliated Hospital of China Medical University, Shenyang, 110001 China; 2 Department of Biochemistry and Molecular Biology, College of Basic Medicine, China Medical University, Shenyang, 110001 China; 3 Clinical Cancer Institute, Kanagawa Cancer Center, Yokohama, 241-0815 Japan

**Keywords:** Ovarian cancer, REG4, Aggressive phenotypes, Pathobiological behavior, Prognosis

## Abstract

**Background:**

Although its biological function remains poorly understood, REG4 is reported to be a potent activator of the EGFR/Akt/AP-1 signaling pathway in colon cancer cells and closely linked with the inhibition of apoptosis.

**Methods:**

SKOV3 cells were transfected with a REG4-expressing plasmid or treated with recombinant REG4. We then analyzed proliferation, cell cycle, apoptosis, invasion and metastasis or expression of related molecules. *REG4* expression was examined in normal ovarian tissue, benign and borderline tumors, and cancers by immunohistochemistry or real-time PCR.

**Results:**

REG4 overexpression and the recombinant protein inhibited cell apoptosis, enhanced G_2_/S progression, proliferation, migration and invasion. Furthermore, expression of *Wnt5a*, *p70s6k, survivin* and *VEGF* expression was increased*,* while *Bax* expression was decreased at both the mRNA and protein levels compared to control or mock cells (*P* < 0.05). *REG4* mRNA levels were higher in benign tumors and primary cancer compared to those in normal ovarian tissue (*P* < 0.05) while, REG4 protein expression was higher in all three tumor types than that in normal ovarian tissue (*P* < 0.05). Higher REG4 mRNA expression was observed in mucinous carcinomas than serous carcinomas (*P* < 0.05), and in well- and moderately-differentiated carcinomas than poorly-differentiated carcinomas (*P* < 0.05). Survival analysis revealed an inverse relationship between REG4 expression and cumulative or relapse-free survival rates of the patients with ovarian cancer as an independent factor (*P* < 0.05).

**Conclusions:**

Our findings indicate that aberrant REG4 expression plays an essential role in early ovarian carcinogenesis and is closely linked to mucinous ovarian tumors, differentiation and adverse prognosis of ovarian cancer by modulating proliferation, apoptosis, migration and invasion.

**Electronic supplementary material:**

The online version of this article (doi:10.1186/s12885-015-1435-2) contains supplementary material, which is available to authorized users.

## Background

Ovarian cancer is one of the leading causes of death due to cancer in women worldwide. The 5-year survival rate for all stages of ovarian cancer is 35–38 %. Due to the lack of effective methods for early diagnosis, most ovarian cancers are only diagnosed at an advanced stage. As such, it is a serious threat to women’s health. Ovarian cancers are believed to originate in the ovarian epithelium, with risk factors such as genetic mutations and family history [1, 2]. An improved understanding of the changes in gene expression and associated molecular mechanisms during ovarian carcinogenesis, as well as identification of novel biomarkers and targets for diagnosis and treatment may result in improvements in diagnosis, treatment and prevention of ovarian cancer.

Regenerating (REG) protein is induced during regeneration of pancreatic islets and belongs to the calcium-dependent lectin (C-type lectin) gene superfamily. It encodes a group of small multifunctional secretory proteins which function as acute phase reactants, lectins, anti-apoptotic factors and growth agents, and include growth factors for pancreatic β-cells, neural cells and epithelial cells in the digestive system. These proteins are primarily involved in cell proliferation and differentiation, inflammation, diabetes and carcinogenesis. To date, three subtypes of the *REG* gene have been identified in humans: *REG1 (1α* and *1β*); *REG3* (*III* and *HIP/PAP*); and *REG4* [3–5]. *REG4*, which is located on human chromosome 1q12–q21, with three variants produced by alternative splicing [6], was identified by high-throughput sequencing of a cDNA library from ulcerative colitis tissues, implying that it has a central role in initiating the multi-step process of colorectal carcinogenesis.

Studies in colon cancer cell lines showed that REG4 expression was enhanced by stimulation with transforming growth factor (TGF)α, EGF, bFGF and HGF [7], and in hepatocellular carcinoma cell lines by stimulation with TGF-β [8]. Furthermore, REG4 has been reported to be a potent activator of the epidermal growth factor receptor/Akt kinase/activator protein-1 (EGFR/Akt/AP-1) signaling pathway, leading to increased expression of Bcl-2, Bcl-xl and survivin, which are associated with apoptosis inhibition [9]. Treatment with recombinant REG4 protein protected normal intestinal crypt cells from ionizing radiation (IR)-induced apoptosis by increasing expression of Bcl-2, Bcl-xL and survivin; whereas overexpression of REG4 in human colorectal cancer (CRC) cells was associated with increased resistance to IR-induced apoptosis. REG4 has also been found to induce expression of matrix metalloproteinase-7, which is involved in liver metastasis [10]. Naito et al. [11] suggested that nuclear CDX2 regulates *REG4* transcription by binding to its 5′-flanking region. Li et al. [12] reported that proteoglycan from *Phellinus linteus* might suppress CRC cells by inhibiting the REG4/EGFR/Akt signaling pathway.

In our previous study, REG4 mRNA and protein expression levels were found to be increased in gastric intestinal metaplasia compared to those in gastric cancer and normal mucosa; higher serum REG4 levels were observed in patients with gastric cancer compared to healthy individuals; and REG4 was expressed more frequently in signet ring cell carcinoma, which closely correlated with MUC-2, and MUC-5 AC expression [13]. Expression of REG4 in CRC cells was significantly lower than in adjacent non-neoplastic mucosa or adenoma, inversely correlated with poor differentiation and venous invasion, and was positively linked to MUC2 expression and EGFR phosphorylation at Tyr1068 [14]. Wang et al. [15] found that expression of both REG4 mRNA and protein in glioma tissues was significantly higher than that in corresponding non-neoplastic brain tissues, and that increased expression of REG4 was significantly associated with advanced pathological grade and low Karnofsky performance score of gliomas. Here, we investigated the effect of REG4 overexpression and recombinant REG4 on the proliferation, apoptosis, invasion and migration of ovarian cancer cell lines, and explored the related mechanisms. In addition, we examined REG4 expression in normal ovarian tissue, benign and borderline ovarian tumors, and primary and metastatic cancers, and compared our findings with the clinicopathological and prognostic parameters of tumors surgically resected from the patients with ovarian cancer.

## Methods

### Cell culture and transfection

Ovarian cancer cell lines: CAOV-3 (serous adenocarcinoma); OVCAR3 (serous cystadenocarcinoma); SKOV3 (papillary serous cystadenocarcinoma); HO8910 (serous cystadenocarcinoma); HO8910-PM (invasive adenocarcinoma); and ES-2 (clear cell carcinoma) were purchased from ATCC (Manassas, VA, USA), SKOV3/DDP (cisplatin-resistant SKOV3) was purchased from the Tumor Cell Bank of Chinese Academy of Medical Science (Peking, China). H08910, H08910-PM, OVCAR3 and SKOV3/DDP were maintained in RPMI 1640; CAOV3 was maintained in DMEM; ES-2 and SKOV3 were maintained in McCoy’s 5A, all mediums were supplemented with 10 % fetal bovine serum (FBS), 100 U/mL penicillin and 100 μg/mL streptomycin, in a humidified atmosphere of 5 % CO_2_ at 37 °C. The cells were seeded in dishes 24 h before transfection with *pCI-REG4* (kindly donated by Prof. Akira Sugawara; Department of Advanced Biological Sciences for Regeneration, Tohoku University, Japan). Cells were treated with recombinant REG4 (BioVendor, Asheville, NC, USA), or transfected with *pCI-REG4* (SKOV3/REG4) or pCI vector (Mock). Transfected cells were selected using 500 mg/L G418 according to the manufacturer’s instructions (QIAGEN, Valencia, CA, USA).

### Cell viability assay

Briefly, 2.0 × 10^3^ cells/well was seeded on 96-well plates. After cells were adhered at 37 °C in an atmosphere of 5 % CO_2_, 10 μL of CCK-8 (Cell Counting Kit-8; Dojindo, Mashikimachi, Japan) solution was added to each well at different time-points (0 h, 12 h, 24 h, and 48 h) and the plates were incubated for a further 3 h. The number of viable cells was counted by measuring the absorbance at 450 nm using a microplate reader.

### Flow cytometric cell cycle analysis

After incubation at 37 °C in an atmosphere of 5 % CO_2_ for 48 h, cells were detached by trypsinization, collected, washed twice with PBS and fixed in 5 mL ice-cold ethanol for at least 2 h. The cells were again washed twice with PBS and incubated with 500 μL RNase (0.25 mg/mL) for 30 min at 37 °C. Cells were pelleted and resuspended in propidium iodide (PI) at a concentration of 50 μg/mL and incubated in the dark for 30 min at 4 °C. Cell cycle analysis was performed by analyzing PI staining levels by flow cytometry.

### Flow cytometric apoptosis assay

Cell apoptosis was determined by PI and Annexin V-FITC staining (KeyGEN Biotech, Nanjing, China). In brief, cells were incubated for 48 h, washed twice with ice-cold PBS, resuspended at a density of 1 × 10^6^ cells/mL in 1× Binding Buffer and then incubated with 200 μL 1× Binding Buffer containing 10 μL FITC-Annexin V. Samples were gently vortexed and incubated for 15 min at 25 °C in the dark, then 300 μL 1× Binding Buffer and 5 μL PI was added to each tube. Samples were gently vortexed and incubated for a maximum of 1 h at 25 °C in the dark. Flow cytometry was performed within 1 h of incubation.

### Wound healing assay

Cells were seeded at a density of 1.0 × 10^6^ cells/well in 6-well culture plates. After reaching confluence, each cell monolayer was scraped with a 200 μL pipette tip to create a scratch, washed three times with PBS, and cultured in FBS-free medium. Cells were photographed at 0, 12, 24, and 48 h (*n* = 3) and the scratch area was measured using Image software (National Institutes of Health, Bethesda, MD, USA). The wound healing rate = (area of original wound-area of actual wound at different times)/ area of original wound × 100 %.

### Cell invasion assays

Cells (5 × 10^5^) were seeded in the top chamber of Matrigel-coated transwell plates (BD Bioscience, San Jose, CA, USA) with serum-free McCoy’s 5A medium. The lower compartment of the chamber contained 10 % v/v FBS as a chemoattractant. After incubation for 48 h, cells on the membrane were scrubbed, washed with PBS, fixed in 100 % methanol and stained with Crystal violet dye to measure invasion.

### Selection of patient tissue samples

Samples of normal ovarian tissue, ovarian epithelial benign tumors (serous cystadenoma and mucinous cystadenoma) and borderline tumors, primary and metastatic cancers in the omentum were collected from surgical resections between January 2003 and December 2011 at Department of Gynecology, The First Affiliated Hospital of China Medical University, Shenyang, China. The average age at surgery was 51.2 years (range 20–81 years). The median age (56 years) was chosen as the divider in Table 2 and Additional file 1: Table S2. The majority of samples were routinely prepared for storage in pathological blocks; the remaining samples were frozen immediately in liquid nitrogen and stored at −80 °C until required. In total, 123 samples were fresh-frozen for RT-PCR analysis, while 337 samples were fixed in paraffin for immunostaining analysis (details can be found in Additional file 1: Table S1). Each ovarian cancer was staged according to the International Federation of Gynecology and Obstetrics (FIGO) staging system and histology was defined according to World Health Organization (WHO) classification system. Classification of the differentiation classes of the samples (HE staining) was evaluated by experienced physiologists according to the tumor cell differentiation grades. None of the patients had undergone chemotherapy, radiotherapy or adjuvant treatment prior to surgery. Patients were followed up by consulting their case documents and by telephone. Written informed consent was provided by each participant. The study protocols conformed to the standards set by the Declaration of Helsinki and were approved by China Medical University Ethics Committee.

### Western blot analysis

Protein was extracted in RIPA lysis buffer and its concentration was determined using a protein assay kit (Bio-Rad, Hercules, CA, USA). The denatured protein was separated by 10 % sodium dodecyl sulfate-polyacrylamide gel electrophoresis (10 % SDS-PAGE) and transferred to a Hybond membrane. The membranes were blocked overnight in 5 % skimmed milk in Tris-buffered saline with Tween 20 (TBST, 10 mM Tris-HCl, 150 mM NaCl, 0.1 % Tween 20). For immunoblotting, the membranes were incubated for 1 h with antibodies against the following proteins: REG4 (RD Systems Inc, Minneapolis, MN, USA); Wnt5a (Santa Cruz Biotechnology, Dallas, TX, USA); phosphorylated p70 s6k (T421/s424; Cell Signaling, Danvers, MA, USA); Bax (Santa Cruz Biotechnology); survivin (Santa Cruz Biotechnology); and VEGF (Santa Cruz Biotechnology). The membranes were then rinsed with TBST and incubated with IgG conjugated to horseradish peroxidase (HRP; Dako, Carpinteria CA, USA) for 1 h. The bands were visualized using a Fuji 4000 imaging system (Fuji, Tokyo, Japan) after applying electrochemiluminescent (ECL) detection reagents (Santa Cruz Biotechnology). After detection, the membranes were washed with Western blot (WB) Stripping Solution (pH2-3, Nacalai, Tokyo, Japan) for 20 min and treated, as described above, using mouse GAPDH antibody (Sigma-Aldrich St. Louis, MO, USA) as an internal control.

### Real-time reverse transcriptase-polymerase chain reaction (real-time RT-PCR)

Total RNA was extracted from the cell lines using QIAGEN RNeasy mini kit (QIAGEN) according to the manufacturer’s protocol; then 2 μg of total RNA was used to synthesize cDNA using AMV reverse transcriptase and random primer (Takara, Ostu, Japan) and then subjected to real-time PCR examination using the SYBR Premix Ex Taq™ II kit (Takara) according to the manufacturer’s protocol. The oligonucleotide primers for RT-PCR were synthesized by Takara and are listed in Additional file 1: Table S4.

### Immunohistochemistry (IHC)

Tissue samples were deparaffinized with xylene, rehydrated with alcohol, and subjected to antigen retrieval in target retrieval solution (TRS; Dako) for 15 min by microwave oven irradiation (Oriental Rotor Ltd. Co., Tokyo, Japan). Non-specific binding was prevented in 5 % bovine serum albumin (BSA) for 5 min. The sections were incubated with goat anti-human REG4 antibody (1:50) or rabbit-anti-(human) Ki-67 antibody (1:50; Dako) for 15 min and then treated with anti-goat or anti-rabbit conjugated to HRP (1:100; Dako) for 15 min. After each treatment, the slides were washed three times with TBST for 1 min, and the proteins were visualized with 3, 3′-diaminobenzidine (DAB). All incubations were performed in a microwave oven, to allow intermittent irradiation, as previously described [16]. After counterstained with Mayer’s hematoxylin, the sections were dehydrated, cleared and mounted. Negative controls were prepared by omitting the primary antibody.

One hundred cells were randomly selected and counted from five representative fields of each section by three independent observers (CS, YZ, and ZHC). Any inconsistencies were checked by three observers until a final agreement was reached. Positive expression was graded as follows: 0 = negative; 1 = 1–49 %; 2 = 50–74 %; 3 ≥ 75 %. The staining intensity score was graded as follows: 1 = weak; 2 = intermediate; 3 = strong. The grade and score for REG4 or Ki-67 were multiplied to obtain a final expression score as follows: − = 0; + = 1–2; ++ = 3–4; +++ = 6–9.

### Measurement of cancer antigen 125 (CA-125)

Quantitative Chemiluminescence Immunoassay kits (Gentaur, Paris, France) were used to detect serum CA-125. Briefly, 50 μl of standard (0–1000 U/mL) specimen or control samples were dispensed into appropriate wells in a microtiter plate. Enzyme conjugate reagent (100 μL) was added to each well and gently mixed, and the plate was incubated for 60 min at room temperature. The wells were rinsed and flicked with wash buffer and residual water droplets were removed by striking the well sharply onto absorbent paper. Finally, chemiluminescence substrate solution (100 μL) was added to each well, mixed gently and the absorbance was measured.

### Statistical analysis

Statistical analysis was performed using SPSS v. 10.0 software. Spearman’s rank correlation coefficient was used to analyze rank data, and Mann-Whitney U-test was used to differentiate the means between different groups. Kaplan-Meier survival plots were generated and comparisons between survival curves were made using Log-rank statistics. Multivariate analysis of parameters such as age, pathological classification, FIGO staging, differentiation and Ki-67 expression was carried out using Cox’s proportional hazard model. A *P*-value <0.05 was considered to indicate statistical significance.

## Results

**Fig. 1:**
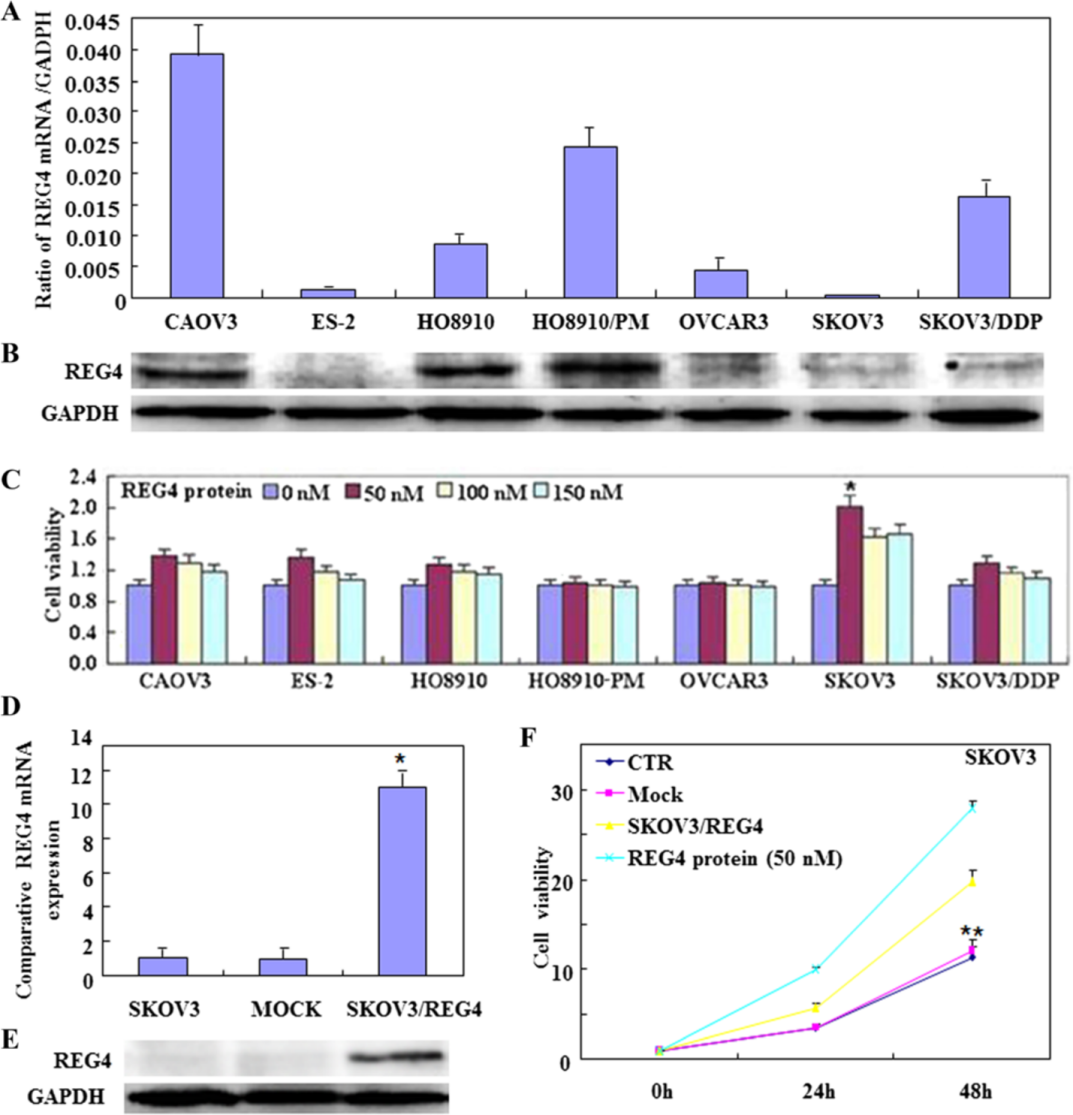
The effects of REG4 overexpression and the recombinant protein on ovarian cancer cells. Expression levels of *REG4* mRNA and protein in ovarian cancer cell lines (CAOV3, ES-2, HO8910, HO8910-PM, OVCAR3, SKOV3 and SKOV3/DDP) were screened by (**a**, *n* = 3) real-time RT-PCR and (**b**, respectively) Western blotting. After treatment with the recombinant protein (50 nM), growth of SKOV3 cells was significantly increased (**c**, *n* = 3), and ectopic REG4 overexpression in SKOV3 cells was observed by (**d**, *n* = 3) real-time RT-PCR and (**e**, respectively) Western blotting. The treatment with the *REG4*-expressing plasmid or the recombinant protein enhances (**f**, *n* = 3) cell proliferation, compared with mock or control cells. Results are expressed as mean ± SD of three separate experiments; *P < 0.05

**Fig. 2:**
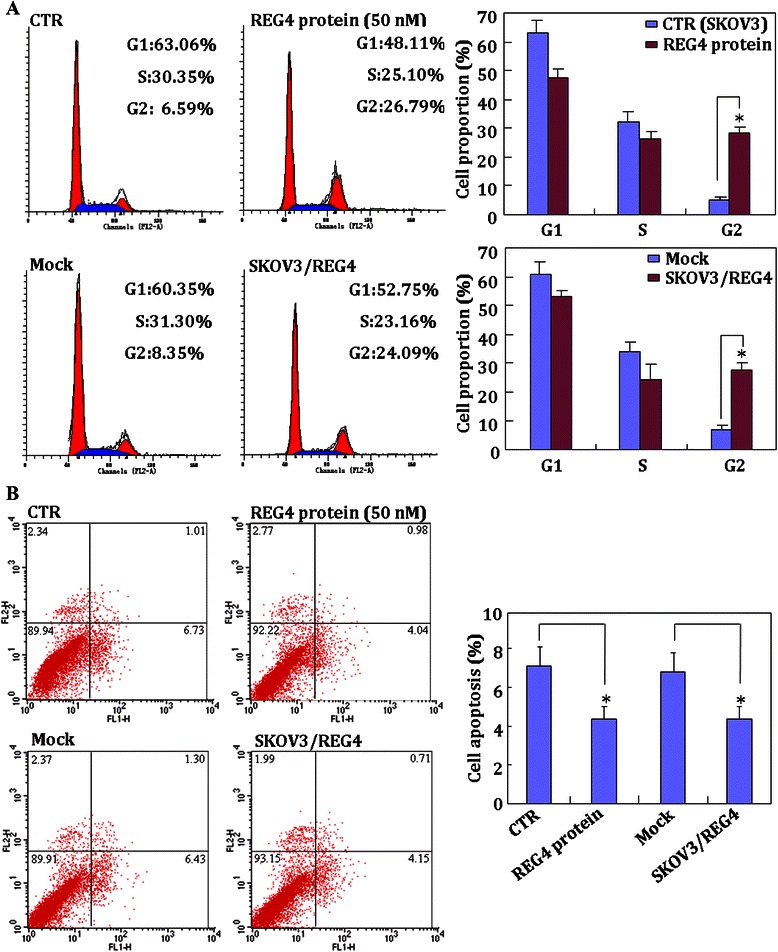
Effects of *REG4* on the cell cycle and cell apoptosis of ovarian cancer cells. The treatment with the *REG4*-expressing plasmid or its recombinant protein enhances (**a**, *n* = 3) G_2_/S progression, (**b**, *n* = 3) but suppresses apoptosis, compared with control or mock cells. Results are expressed as mean ± SD of three separate experiments; *P < 0.05

**Fig. 3:**
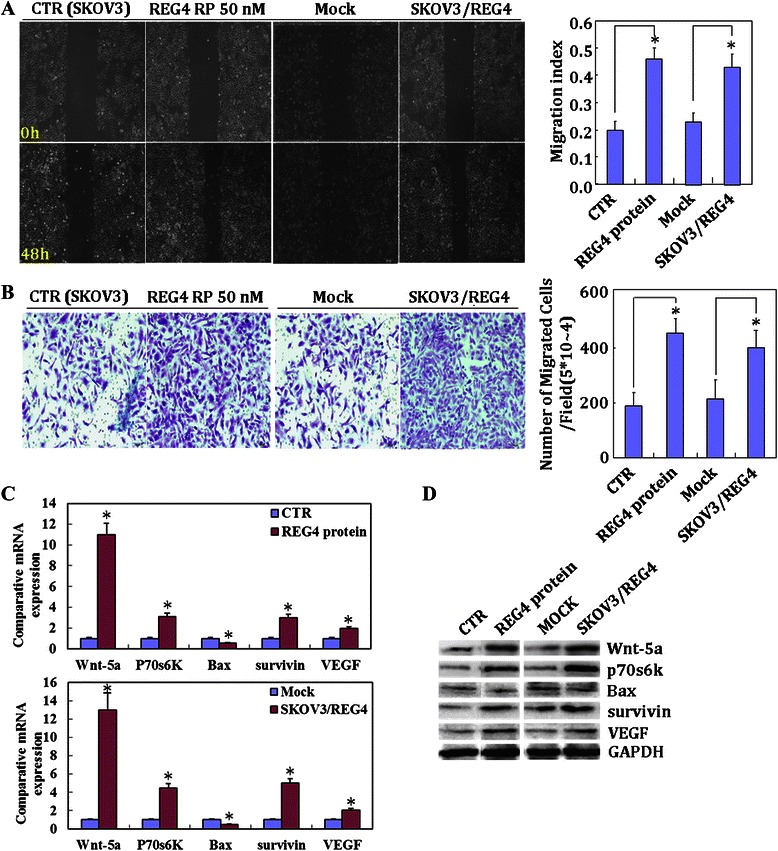
Effects of *REG4* on the invasive and metastatic ability of ovarian cancer cells. The treatment with the *REG4*-expressing plasmid or the recombinant protein induces migration in wound healing assays (**a**, *n* = 3), and invasion in Matrigel transwell assays (**b**, *n* = 3) compared with the control and mock cells. In addition, expression of *Wnt5a*, *p70s6k, survivin* and *VEGF* is increased at both mRNA and protein levels in SKOV3, as observed by (**c**) real-time RT-PCR and (**d**) Western blotting, respectively; however, *Bax* expression was decreased. Results are expressed as mean ± SD of three separate experiments; *P < 0.05

*REG4* mRNA was found to be expressed at a high level in CAOV3, SKOV3/DDP, HO8910, HO8910-PM cell lines compared with those in ES-2, SKOV3 and OVCAR3 cell lines (Fig. 1a). REG4 protein was expressed at a high level in CAOV3, HO8910 and HO8910-PM cells compared with those in the other cell lines (Fig. 1b). In addition, after treatment with recombinant protein REG4 (50 nM), SKOV3 cell growth was significantly increased (Fig. 1c), compared with that of normal SKOV3 cells (CTR). To elucidate the role of REG4 in ovarian cancer, SKOV3 cells were transfected with a *REG4*-expressing plasmid and analyzed by real-time RT-PCR (Fig. 1d) and Western blotting (Fig. 1e). Subsequent assays compared the treated SKOV3 cells to control or mock cells (cells transfected with pCI-REG4-Mut plasmid) and showed significant increases in cell growth (proliferation assay; *P* < 0.05; Fig. 1f); G_2_/S progression (flow cytometric cell cycle analysis; *P* < 0.05; Fig. 2a); and apoptosis-inhibition (flow cytometric apoptosis assay; *P* < 0.05; Fig. 2b). Furthermore, there was an increase in migration (wound healing assay; Fig. 3a) and invasion (Transwell cell migration assay; Fig. 3b). Real-time RT-PCR and Western blotting also showed increased expression of Wnt5a, p70s6k, survivin and VEGF, in *REG4*- transfectants or cells treated with recombinant protein compared to control and mock cells, while the converse was observed for Bax expression (Fig. 3c and d).

**Fig. 4:**
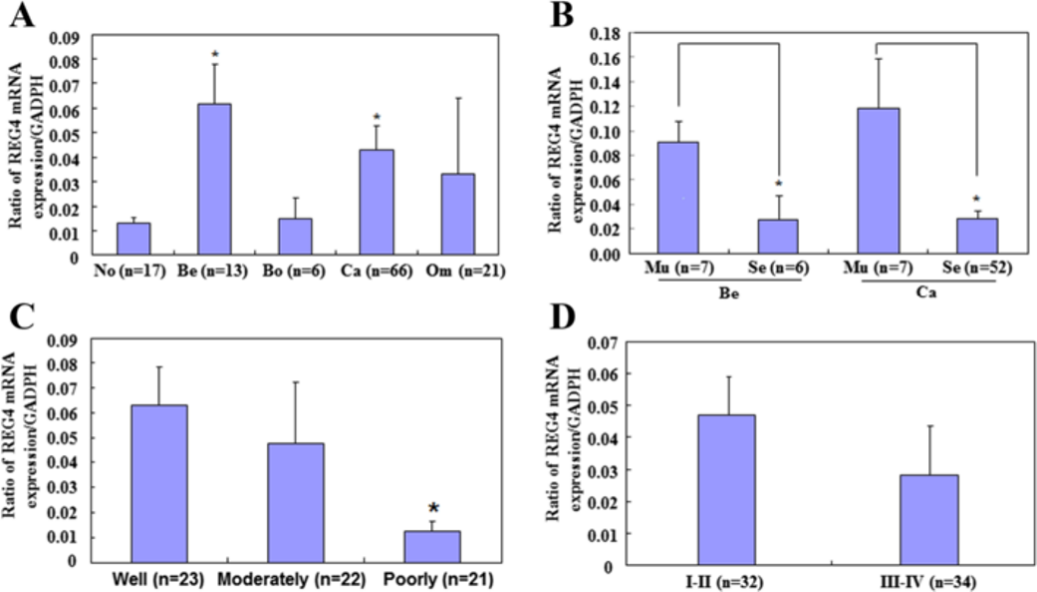
Relationship between *REG4* mRNA expression and clinicopathological features of ovarian cancer. *REG4* mRNA expression was quantified by real-time RT-PCR in normal ovarian tissue (No; *n* = 17), benign (Be; *n* = 10) and borderline (Bo; *n* = 6) tumors, primary (Ca; *n* = 66) and metastatic cancers in the omentum (Om; *n* = 21). **a***REG4* mRNA expression levels are significantly higher in both benign ovarian tumors and primary carcinoma compared to normal ovarian tissue. **b** Mucinous (Mu) tumors show higher *REG4* mRNA expression levels than serous (Se) tumors; and Mann-Whitney U test showed that *REG4* mRNA expression is higher in well- and moderately-differentiated tumors than in poorly-differentiated tumors (**c**), but there was no significant difference between (**d**) FIGO staging I–II & III–IV; *P < 0.05

As determined by real-time RT-PCR, *REG4* mRNA levels were higher in benign ovarian tumors than those in normal ovarian tissue (*P* < 0.001; Fig. 4a); and higher in primary carcinoma than in normal ovarian tissue (*P* = 0.048; Fig. 4a). Expression of *REG4* mRNA was also higher in mucinous benign tumors than serous benign tumors (*P* = 0.03; Fig. 4b), and in mucinous carcinoma than serous carcinoma (*P* < 0.001; Fig. 4b), and in well- and moderately-differentiated compared to poorly-differentiated carcinomas (*P* = 0.011; Fig. 4c); however, there was no correlation between *REG4* mRNA expression and FIGO staging (R^2 = 0.022, *P* = 0.225; Fig. 4d), and between *REG4* mRNA expression and age (*P =* 0.06; Additional file 1: Table S2).

**Fig. 5:**
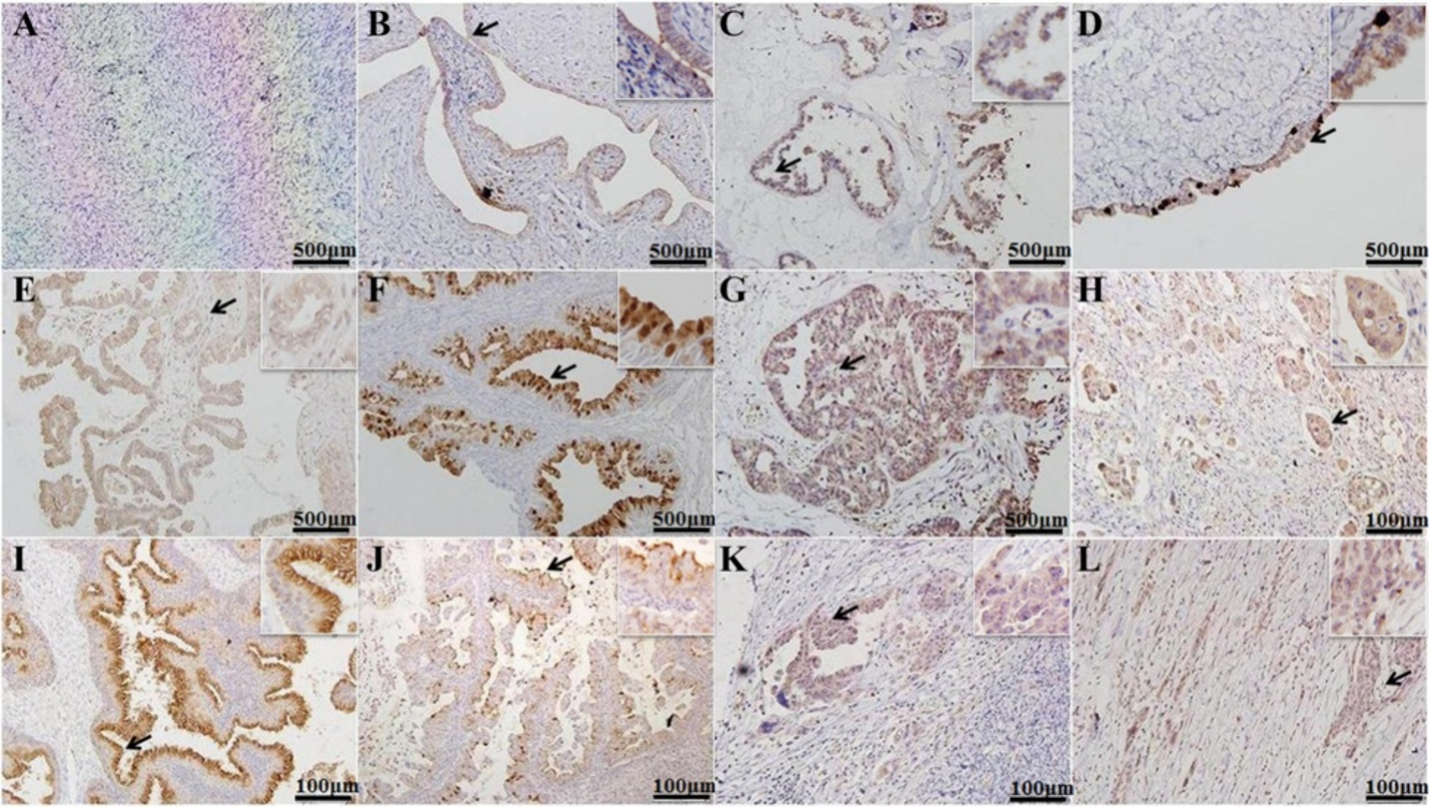
Immunohistochemistry of REG4 protein expression in ovarian carcinogenesis. The micrographs show that REG4 protein is not expressed in the cytoplasm of fiber cells of normal ovary tissue (**a**) and only weakly in the cytoplasm of fallopian tube cells (**b**), but it is strongly expressed in the cytoplasm of serous and mucinous adenoma (**c**, **d**); serous and mucinous borderline tumor (**e**, **f**); serous (**g**, **h**) and mucinous (**i**, **j**) adenocarcinoma; and adenocarcinoma in the omentum (**k**, **l**)

Immunostaining revealed that REG4 was strongly expressed in the cytoplasm of serous and mucinous adenoma, serous and mucinous borderline tumors, serous and mucinous adenocarcinoma, and adenocarcinoma in the omentum; but was not expressed in the fiber cells of normal ovarian tissue and only very weakly in the fallopian tube (Fig. 5). As shown in Table 1, REG4 protein expression was detectable in normal ovarian tissue (23.1 %, 6/26), benign ovarian tumors (70 %, 7/10), borderline ovarian tumors (81.8 %, 18/22), primary cancer (50.6 %, 119/235) and metastatic carcinoma in the omentum (61.4 %, 27/44). REG4 expression was significantly higher in benign tumors, borderline tumors and primary cancer than that in normal ovarian tissue (*P* < 0.05; Table 1), while there was no significant difference between primary and metastatic cancers in the omentum (*P =* 0.076; Table 1). In addition, mucinous carcinomas showed higher REG4 expression than serous ones (*P* < 0.05; Table 2). REG4 expression was significantly higher in well- and moderately- differentiated tumors than in poorly-differentiated ones (*P* = 0.009), and in patients aged ≥ 56 years than in those aged < 56 years (*P* = 0.03), although there were no significant correlation with FIGO staging or expression of the proliferation marker, Ki-67 (*P* > 0.05). In addition, Spearman’s rank correlation coefficient showed no correlation between REG4 protein expression and different sample years (*r* = 0.0061; *P* = 0.355; Additional file 1: Table S3).

**Table 1:** REG4 protein expression during ovarian epithelial carcinogenesis

Groups	n	REG4 protein expression				
		−	+	++	+++	PR (%)
Normal ovarian tissue	26	20	2	2	2	23.1*
Benign ovarian tumor	10	3	2	1	4	70.0
Borderline ovarian tumor	22	4	2	2	14	81.8**
Ovarian cancer	235	116	45	41	33	50.6
Metastatic cancer in omentum	44	17	9	12	6	61.4
Groups	n	REG4 protein expression				
		−	+	++	+++	PR (%)
Primary ovarian cancer	34	7	9	10	8	79.4^#^
Metastatic cancer in omentum	34	12	9	10	3	64.7

PR, positive rate

*P < 0.05 (normal ovarian tissue compared with benign tumor and primary carcinoma)

**P < 0.01 (ovarian borderline tumor compared with normal ovarian tissue)

^#^*P* = 0.076 (metastatic lesions compared with relative primary cancer tissues)

**Table 2:** Relationship between REG4 protein expression and clinicopathological features of ovarian cancers

Clinicopathological features	n	REG4 protein expression					
		−	+	++	+++	PR (%)	*P*-value
Age (years)							0.03*
<56	115	63	25	15	12	45.2	
≥56	120	53	20	26	21	55.8	
Pathological classification							0.009**
Mucinous adenocarcinoma	26	7	6	3	10	73.1*	
Serous adenocarcinoma	174	93	29	35	17	46.6	
Miscellaneous subtypes	35	16	10	3	6	54.3	
FIGO staging							0.485
I–II	87	47	15	10	15	46.0	
III–IV	148	69	30	31	18	53.4	
Differentiation							0.001***
Well-differentiated	59	21	15	11	12	64.4	
Moderately-differentiated	89	39	17	19	14	56.2	
Poorly-differentiated	87	56	13	11	7	35.6	
Ki-67 expression							0.419
−	56	32	10	7	7	42.9	
+	34	13	7	10	4	61.8	
++	34	18	6	7	3	47.1	
+++	33	16	7	6	4	51.5	

*P < 0.05, which were pointed out by bold text, indicate statistically significant

**P < 0.01 (Mucinous adenocarcinoma compared with other pathological subtypes)

***P = 0.001 (Well- and moderately-differentiated compared with poorly-differentiated)

**Fig. 6:**
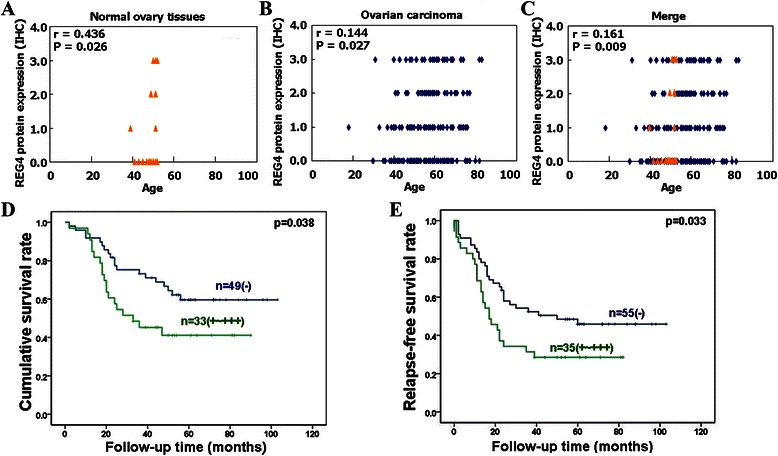
The prognostic significance of REG4 protein expression in ovarian cancer. Spearman’s rank correlation test showed significant correlation between REG4 protein expression in normal ovarian tissues and patient age (*r* = 0.436, *P* = 0.026, Fig. 6**a**), and between ovarian carcinomas and patient age (*r* = 0.144, *P* = 0.027, Fig. 6**b**). When combined together, REG4 expression was still correlated with patient age (*r* = 0.0161, *P* = 0.09, Fig. 6**c**). Kaplan-Meier survival curves (**d**, **e**) indicate a negative association between REG4 protein expression and cumulative or relapse-free survival rates of patients with ovarian cancer. REG4 protein expression was based on the IHC results classified as: 0 (negative), 1 (+), 2 (++), and 3 (+++)

Follow-up information was available for 90 patients with ovarian cancer for periods ranging (8 lost to follow-up) from 1–103 months (median, 48 months). Survival curves for the patients with ovarian cancer were stratified according to REG4 protein expression, based on the IHC results (Fig. 6d & e) and subsequent univariate analysis using Kaplan-Meier method indicated an inverse relationship between REG4 expression and cumulative (*P* = 0.038) or relapse-free survival rate (*P* = 0.033). Multivariate analysis using Cox’s proportional hazard model indicated that FIGO staging (*P* = 0.038), differentiation (*P* = 0.007) and REG4 expression (*P* = 0.006) were independent prognostic factors for overall survival of the patients with ovarian cancer (Table 3); whereas patient age, remnant foci size, pathological classification or serum CA-125 levels were not (*P* > 0.05; Table 3). Of all these parameters, only REG4 expression was found to be an independent prognostic factor of relapse-free survival of the patients with ovarian cancer (*P* = 0.013), while patient age, pathological classification, degree of differentiation, remnant foci size, serum CA-125 level and FIGO staging were not (*P* > 0.05, Table 3).

**Table 3:** Multivariate analysis of clinicopathological parameters with the overall survival of patients with ovarian cancer

Clinicopathological parameters	Cumulative survival		Relapse-free survival	
	Relative risk (95 % CI)	*P-*value	Relative risk (95 % CI)	*P-*value
Age (≥56 years)	1.926 (0.916–4.050)	0.084	1.784 (0.963–3.306)	0.066
Pathological subtypes (serous carcinoma)	0.854 (0.329–2.221)	0.747	0.731 (0.329–1.625)	0.442
FIGO staging (III–IV)	2.762 (1.058–7.214)	**0.038**	1.619 (0.745–3.520)	0.224
Differentiation degree (poor)	3.007 (1.350–6.700)	**0.007**	1.892 (0.988–3.622)	0.054
REG4 expression (+ to +++)	3.057 (1.369–6.827)	**0.006**	2.282 (1.189–4.380)	**0.013**
Remnant foci size (≥1 cm)	2.343 (0.995–5.519)	0.051	1.834 (0.869–3.872)	0.112
Serum CA-125 level (≥500)	0.813 (0.402–1.645)	0.564	1.004 (0.556–1.812)	0.989

*Abbreviation*: *CI* = confidence interval

*P* < 0.05, which were pointed out by bold text, indicate statistically significant

## Discussion

Previous reports have shown that *REG4* mRNA is strongly expressed in inflamed epithelium, dysplasia and cancerous lesions of ulcerative colitis [17], and REG4 overexpression has been observed in gastric, colorectal, pancreatic, hepatocellular and prostate cancers [5, 9–14]. Here, real-time PCR showed that *REG4* mRNA was overexpressed in both benign ovarian tumors and cancers. Furthermore, immunohistochemistry showed that REG4 expression was increased in benign tumors, borderline tumors and primary carcinoma compared to normal ovarian tissue. This was consistent with our previous observations in gastric [13] and colorectal [14] cancers. Taken together, these observations suggest that REG4 overexpression plays an essential role as an early event in ovarian carcinogenesis.

We found that REG4 mRNA and protein expression levels in ovarian cancer were significantly higher in mucinous tumors than in serous tumors, and were positively associated with differentiation. Again, this is consistent with our previous findings in gastric cancer, which showed positive REG4 expression in signet ring cells, and mucinous carcinoma, and higher expression in well- and moderately-differentiated adenocarcinoma than poorly-differentiated adenocarcinoma [13]. In addition, REG4 mRNA expression was higher in mucinous benign tumors and carcinomas compared to relative serous subtypes, while there was no significant difference in REG4 mRNA expression between mucinous benign tumors and mucinous carcinomas, and between serous benign tumors and serous carcinomas. Huang et al. reported that REG4 expression was also overexpressed in mucinous borderline tumors and primary mucinous carcinomas [18]. Taken together, these results suggest that REG4 overexpression is closely linked to the pathogenesis of ovarian mucinous carcinoma and differentiation of ovarian cancer. Furthermore, our observations revealed no significant difference in *REG4* mRNA or protein expression between stages I–II and III–IV ovarian cancer, which was also consistent with our previous findings in gastric cancer [13]. Conversely, primary prostate cancers (PCa) have been reported to express low levels of *REG4* mRNA, whereas the majority of metastatic PCa tumors expressed high levels [19], and REG4 expression has been associated with lymph node metastasis, recurrence of liver metastasis and tumor stage in colorectal carcinoma [7, 20]. *REG4* mRNA expression levels in surgically resected specimens and the peritoneal wash were closely related to those in wall-penetrating gastric carcinoma.

Recently, secretory REG4 protein was found to act as a potent activator of the EGFR/Akt/AP-1 signaling pathway, through phosphorylation of EGFR [9, 21]. To elucidate the role and molecular mechanisms of REG4 in ovarian cancer cells, SKOV3 cells were transfected with *REG4*-expressing plasmids or exposed to recombinant REG4. We found that G2/S progression, apoptotic inhibition, proliferation, migration and invasion were significantly increased. Further investigation showed that both treatments increased expression of *Wnt5a*, *p70s6k, survivin* and *VEGF* at both the mRNA and proteins levels, but *Bax* expression was decreased. *Wnt5a*, *p70s6k, survivin, VEGF* and *Bax* are involved in the EGFR signaling pathway, apoptosis, invasion and metastasis in malignancies. Therefore, these findings suggest that increased REG4 protein expression, or its ectopic overexpression, promotes aggressive behaviors in ovarian cancer cells by modulating expression of phenotype-related genes [22–26]. Our results are supported by reports pf previous *in vitro* and *in vivo* experiments showing that REG4 inhibits apoptosis by increasing the expression of *Bcl-2, Bcl-xl* and *survivin* [9, 10].

Although REG4 has been reported to be significantly correlated with aggressive biological behaviors of malignancies [7, 20], the prognostic relevance of REG4 expression remains controversial. Both previous reports found no relationship between REG4 expression and survival of patients with gastric cancer [13, 24]. In contrast, Ohara et al*.* [20] revealed that REG4 expression was significantly associated with longer relapse-free survival of PCa patients and acted as an independent prognostic factor in PCa. In accordance with previous reports on gliomas [15] and CRC [7], we found an inverse relationship between REG4 expression and cumulative or relapse-free survival rate of patients with ovarian cancer. Our multivariate model indicates that REG4 expression, FIGO staging and differentiation are independent prognostic factors of overall survival in the patients with ovarian cancer. REG4 expression was also shown to be an independent prognostic factor of relapse-free survival.

## Conclusions

REG4 expression was upregulated in precancerous ovarian lesions, then marginally downregulated as the ovarian epithelial cells underwent malignant transformation. Overexpression of REG4 protein increased the aggressiveness of ovarian cancer, as indicated by reduced apoptosis and increased proliferation, migration and invasion. We speculate that this may be correlated with the altered expression of Wnt5a, p70s6k, survivin, VEGF and Bax. Furthermore, REG4 expression was closely linked with mucinous tumors, differentiation and adverse prognosis of ovarian cancer. Taken together, our findings suggest that REG4 represents an independent indicator of poor prognosis in ovarian cancer.

## Additional file

Additional file 1: Table S1. Proportion of the tumor types in different categories. **Table S2.** Relationship between REG4 mRNA expression and clinicopathological features of ovarian carcinomas. **Table S3.** REG4 protein expression during different years among ovarian epithelial carcinogenesis. **Table S4.** Primer sequences used for real-time RT-PCR.

## Abbreviations

REG: Regenerating
P70s6k: p70 ribosomal S6 kinase
VEGF: Vascular endothelial growth factor
GAPDH: Glyceraldehyde-3-phosphate dehydrogenase
TGF: Transforming growth factor
EGFR/Akt/AP-1: Epidermal growth factor receptor/Akt kinase/activator protein-1
EGF: Epidermal growth factor
FGF: Fibroblast growth factor
HGF: Hepatocyte growth factor
MUC-2: Mucin 2, oligomeric mucus/gel-forming
FIGO: The International Federation of Gynecology and Obstetrics
