# Supplementary material for: The role of the REG4 gene and its encoding product in ovarian epithelial carcinoma
Source: BMC Cancer. 2015 Jun 16;15:471. doi: 10.1186/s12885-015-1435-2 (PMC4469329; doi:10.1186/s12885-015-1435-2)
Supplement: Additional file 1: Table S1. — Proportion of the tumor types in different categories. Table S2. Relationship between REG4 mRNA expression and clinicopathological features of ovarian carcinomas. Table S3. REG4 protein expression during different years among ovarian epithelial carcinogenesis. Table S4. Primer sequences used for real-time RT-PCR. [file 12885_2015_1435_MOESM1_ESM.doc]

**Additional file 1**

**Table S1.** Proportion of the tumor types in different categories.

| **Groups** | Fresh-frozen | Paraffin |
| --- | --- | --- |
| Normal ovarian tissue | 17 (13.8%) | 26 (7.7%) |
| Benign ovarian tumor | 13 (10.6%) | 10 (3.0%) |
| Borderline ovarian tumor | 6 (4.9%) | 22 (6.5%) |
| Ovarian carcinoma | 66 (53.7%) | 235 (69.7%) |
| Metastatic carcinoma in omentum | 21 (17.1%) | 44 (13.1%) |
| Number | 123 | 337 |

Fresh-frozen tissues were used for RT-PCR, while Paraffin tissues for IHC

**Table S2**. Relationship between REG4 mRNA expression and clinicopathological features of ovarian carcinomas.

| **Clinicopathological Features** | **n** | **REG4 mRNA expression** | ***P-*value** |
| --- | --- | --- | --- |
| **Ages** |  |  | 0.06 |
| <56 | 33 | 0.0523 ± 0.0745 |  |
| ≥56 | 33 | 0.0248 ±0.0354 |  |
| Pathological classification |  |  |  |
| Mucinous adenocarcinoma | 7 | 0.1190 ± 0.11271 |  |
| Serous adenocarcinoma | 52 | 0.0284 ± 0.0421 | **1.08E-04 **** |
| Miscellaneous subtypes | 7 | 0.0332 ± 0.0460 | **7.02E-05 #** |
| FIGO staging |  |  | 0.225 |
| I-II | 32 | 0.0478 ± 0.0673 |  |
| III-IV | 34 | 0.0299 ± 0.0507 |  |
| Differentiation |  |  |  |
| Well-differentiated | 23 | 0.0636 ± 0.0738 | **0.011 *** |
| Moderately-differentiated | 22 | 0.0321 ± 0.0590 |  |
| Poorly-differentiated | 21 | 0.0178 ± 0.0253 |  |

* *P* = 0.011 (Well- differentiated compared with Poorly- & Moderately-differentiated)

*** P =* **1.08E-04 (**Mucinous adenocarcinoma compared with Serous adenocarcinoma)

# *P* = **7.02E-05** (Mucinous adenocarcinoma compared with Miscellaneous subtypes)

**Table S3.** REG4 protein expression during different years among ovarian epithelial carcinogenesis.

| **Year** | **n** | **REG4 protein expression** | | | | |
| --- | --- | --- | --- | --- | --- | --- |
|  |  | **-** | **+** | **++** | **+++** | **PR (%)** |
| **2003** | 9 | 4 | 2 | 1 | 2 | 55.6 |
| **2004** | 13 | 5 | 4 | 3 | 1 | 61.5 |
| **2005** | 25 | 15 | 4 | 3 | 3 | 40.0 |
| **2006** | 22 | 10 | 5 | 3 | 4 | 54.5 |
| **2007** | 43 | 26 | 7 | 4 | 6 | 39.5 |
| **2008** | 56 | 27 | 8 | 12 | 9 | 51.8 |
| **2009** | 32 | 15 | 8 | 6 | 3 | 53.1 |
| **2010** | 21 | 8 | 4 | 7 | 2 | 61.9 |
| **2011** | 14 | 6 | 3 | 2 | 3 | 57.1 |
| **All** | 235 | 116 | 45 | 41 | 33 | 50.6 |

Spearman’s rank correlation coefficient showed no significant correlation between REG4 protein expression and different years: r = 0.061; p = 0.355.

**Table S4.** Primer sequences used for real-time RT-PCR

| **Names** | **Primer sequence** | **Distribution** | **AT**  **(^o^C)** | **Product**  **Size (bp)** | **Extension**  **Time (s)** |
| --- | --- | --- | --- | --- | --- |
| *REG4* | F: 5’-TAACTTGGAGCAGCAACGAATG-3’  R: 5’-GGCTAGCAGAAAGGAAGAGGA-3’ | NM_032004  682-803 | 60 | 122 | 34 |
| *WNT5A* | F: 5'-GCGAAGACAGGCATCAAA- 3'  R: 5'-CCTTGGCAAAGCGGTAG-3' | NM_003392  946-1240 | 60 | 295 | 34 |
| *RPS6KB1* | F: 5'-TAAAGCATCCCTTCATCG-3'  R: 5'-CAGGCAGTGTCTTCCATAA- 3' | NM_003161  552-690 | 60 | 139 | 34 |
| *BAX* | F: 5'-TGCTTCAGGGTTTCATCCA-3' R: 5'-GACACTCGCTCAGCTTCTTG-3' | NM_138761  146-256 | 60 | 111 | 34 |
| *survivin* | F: 5'-CTTGGCCCAGTGTTTCTT-3'  R: 5'-GCTTCCAGTCCCTCCCT-3' | DQ227257  159-282 | 60 | 124 | 34 |
| *VEGFA* | F:5' AGGAGGAGGGCAGAATC-3'  R: 5'-ATGTGCTGGCCTTGGT-3' | NM_001171630 1131-1388 | 60 | 258 | 34 |
| *GAPDH* | F: 5’-CAATGACCCCTTCATTGACC-3’  R: 5’- TGGAAGATGGTGATGGGATT-3’ | NM_ 002046  201-335 | 60 | 135 | 34 |

AT = annealing temperature
